# Defining compulsive exercise in eating disorders: acknowledging the exercise paradox and exercise obsessions

**DOI:** 10.1186/s40337-019-0238-2

**Published:** 2019-04-04

**Authors:** Solfrid Bratland-Sanda, Therese Fostervold Mathisen, Jorunn Sundgot-Borgen, Jan Harald Rosenvinge

**Affiliations:** 1 Department of sports, physical education and outdoor sciences, University of South-Eastern Norway, Campus Bø, Gullbringvegen 36, 3800 Bø in Telemark, Norway; 2 Department of sports medicine, Norwegian school of sport sciences, Oslo, Norway; 3 Department of Psychology, UiT -The Arctic University of Norway, Tromsø, Norway

## Abstract

Recently Dittmer et al. (JED 6:1–9, 2018). suggested a transdiagnostic definition and a clinical assessment for compulsive exercise in adolescents and adults with eating disorders. In this letter to the editor, we extend the transdiagnostic bridge to the DSM-5-criteria for obsessive-compulsive disorders and hence raise the issue of exercise obsession without compulsive exercise actions. We argue that, at least among persons with bulimia nervosa or binge eating disorders, a belief in the need to exercise to control food, weight and shape, does not necessarily imply that the actual exercise behaviour is excessive in nature. In our opinion, the high scores displayed on compulsive exercise screening instruments is therefore an exercise paradox. This paradox may call attention to the fact that because such obsessions can impair quality of life, they need to be addressed in the clinical evaluation and treatment. Therefore, we suggest adding “exercise obsession” as a fourth subtype of compulsive exercise.

## Proposed definition of compulsive exercise (CE)

Physical activity, including exercise, as a maintenance factor for eating disorders has been labelled as unhealthy, pathological, destructive, excessive, compulsive, and addictive in nature [1]. Such labels connect to normative conceptions of health, frequency of, and motives for physical exercise. This diversity in conceptions has led to confusion and difficulties in reaching a consensus in the field about the nature of compulsive exercise (CE). Hence, we acknowledge Dittmer et al. [2] who have proposed that CE may be defined and assessed by two obligatory criteria (A and B), a third optional criterion (C) and three subtypes (levels) of exercise (Dittmer et al. 2018:3).

A. *CE defined as 1) excessive that the patient feels driven to perform in response to an obsession or according to rules that must be applied rigidly, and 2) the exercise is aimed at preventing some dreaded consequences or at preventing or reducing distress, often based on distorted beliefs about exercise.*
B. *The CE is time-consuming (takes more than 1 h a day), significantly interferes with the person’s daily routine, occupational functioning or social relationship or is continued despite medical injury, illness, or lack of enjoyment.*
C. *At some point the patient has recognized that the CE is excessive or unreasonable.*
D. *Subtypes 1) vigorous exercise, 2) marked increase in daily movement, and 3) motor restlessness.*

The purpose of such criteria is to identify all aspects of CE, to assist the comparison of research findings across studies, and to make sound clinical evaluation of patients who display CE. However, we are concerned that these proposed criteria may not adequately serve such purposes. We therefore suggest development of the criteria to include the concept of exercise obsession.

## The exercise paradox

The proposed criteria and the subtypes ask for *behavioural manifestations*, *motives* to prevent or avoid negative *consequences* of not exercising, and the personal *burdens* of CE. Seeking diagnostic parsimony rather than diversity, we argue that the CE-criteria do not appear as a separate or new clinical condition. Rather, they seem to align with, or be close to, the DSM-5 diagnostic criteria for an obsessive-compulsive disorder (OCD) [3]. It may be that an adaptation of the DSM-5 criteria for OCD is sufficient to identify and manage CE. The alignment with OCD criteria may allow for a further refinement of the understanding and evaluation of CE due to the distinction between obsessions and compulsions. Thus, development of the proposed criteria [2] should aim to clarify the distinction between obsessions (i.e., rigidly applied rules about a “need” to exercise) and compulsions (i.e., the exercise behaviours). Our concern is that the focus on frequency and motives for exercise (i.e. the excessive part of CE) may underestimate the burden from the exercise obsessions, notably among persons with sedentary behaviour. Evidence suggests that the obsessive, but not the excessive, aspect of exercise is related to bulimia nervosa (BN) [4]. Our own research [5, 6] supports this as women with BN or BED may display high scores on CE-instruments despite their objectively assessed low volume of physical activity, which is well below the official recommendations [7]. We have labelled this the “exercise paradox”. For instance, obsessive beliefs about the need to exercise may reflect an intention to maintain a self-image as being healthy, to seek the benefits of a socially admired active lifestyle, to counteract the effects of binging, and/or avoid the shame associated with purging. Yet the individual might not be able to translate such intentions into action. Adding defeat in meeting societal expectations of healthy exercise in line with the official physical activity recommendations [7], the exercise paradox can inflict significant psychological burdens. It is our contention that this paradox adds scientific and clinical value to the proposed criteria for CE.

## Suggestions for the subtype “exercise obsession”

Acknowledging the proposed subtypes as well as the exercise paradox, we propose a fourth subtype covering the obsessive aspects, i.e. “excessive obsessions”. Prevalence rates of exercise obsessions concurrent with sedentary behaviour in eating disorders lack in the existing literature. In our study [6], 142 women with BN or BED had both valid accelerometer-assessed physical activity and completed the Compulsive Exercise Test (CET). Of these, 20% with BN and 6% of BED showed high CET-score (i.e. ≥15) yet sedentary behaviour (i.e. < 150 min/week with physical activity). In persons without excessive exercise, we are concerned about the risk that the nature and burdens of exercise obsessions may be undetected in clinical evaluation and treatment. We cannot exclude the possibility that such risk may lower the overall treatment outcome for these patients. By contrast, this subtype will enable clinicians to evaluate and approach this paradox in treatment. For persons with this subtype of CE the customary response-prevention approach to OCD may be contraindicative, as we do not want to further reduce the lower level of exercise. Rather, the level should be increased with what is appropriate given the patient’s physical and mental health. The volume and type of physical activity should also be synchronized with the progress in reducing the impact of obsessions, and with the overall treatment aim to promote healthy and balanced exercise, and exercise motivated by enjoyment.

## References

[1] Cunningham HE, Pearman S, Brewerton TD (2016). Conceptualizing primary and secondary pathological exercise using available measures of excessive exercise. Int J Eat Disord.

[2] Dittmer N, Jacobi C, Voderholzer U. Compulsive exercise in eating disorders: proposal for a definition and a clinical assessment. J Eat Disord. 2018;6:1–9. 10.1186/s40337-018-0219-x PMC6260729 30505444

[3] APA (2013). Diagnostic and statistical manual of mental disorders (5th ed.).

[4] Adkins EC, Keel PK (2005). Does “excessive” or “compulsive” best describe exercise as a symptom of bulimia nervosa?. IntJ EatDisord.

[5] Mathisen TF, Rosenvinge JH, Friborg O, Pettersen G, Stensrud T, Hansen BH, Underhaug KE, Teinung E, Vrabel K, Svendsen M (2018). Body composition and physical fitness in women with bulimia nervosa or binge-eating disorder. Int J Eat Disord.

[6] Mathisen TF, Bratland-Sanda S, Rosenvinge JH, Friborg O, Pettersen G, Vrabel KA, Sundgot-Borgen J (2018). Treatment effects on compulsive exercise and physical activity in eating disorders. J Eat Disord.

[7] Haskell WL, Lee IM, Pate RR, Powell KE, Blair SN, Franklin BA, Macera CA, Heath GW, Thompson PD, Bauman A (2007). Physical activity and public health: updated recommendation for adults from the American College of Sports Medicine and the American Heart Association. Med SciSports Exerc.

